# Targeting CCR7-KMT2D enhances CAR-T cell efficacy by suppressing therapy-induced senescence in B-cell non-Hodgkin lymphoma

**DOI:** 10.1186/s12916-026-05030-4

**Published:** 2026-06-26

**Authors:** Jinlan Li, Keke Huang, Huiping Wang, Wanjia Chen, Yajie Zhang, Shanyue Jiang, Nengneng Cao, Wanqiu Zhang, Zelin Liu, Jiawen Chen, Dandan Chen, Ziye Yang, Qianshan Tao, Chaohong Liu, Zhimin Zhai, Furun An, Jiyu Wang

**Affiliations:** 1 Department of Hematology, The Second Hospital of Anhui Medical University, Hefei, 230601 Anhui China; 2 Anhui Medical University, Hefei, 230032 Anhui China; 3 Department of Pathogen Biology, School of Basic Medicine, Tongji Medical College, State Key Laboratory for Diagnosis and Treatment of Severe Zoonotic Infectious Disease, Huazhong University of Science and Technology, Wuhan, 430030 Hubei China

**Keywords:** C-C chemokine receptor 7, Cellular senescence, CD19 chimeric antigen receptor T cells, B cell non-Hodgkin’s lymphoma

## Abstract

**Background:**

The efficacy of anti-CD19 Chimeric Antigen Receptor (CAR) T-cell therapy has been demonstrated in patients with relapsed or refractory B-cell non-Hodgkin lymphoma (B-NHL). However, therapy-induced senescence (TIS) has been identified as a novel resistance mechanism, potentially exacerbating immunosuppression and impairing CAR-T cell function.

**Methods:**

Raji and SU-DHL-2 cells were treated with 40 nM doxorubicin (Dox) for 72 h to induce TIS, and CCR7 was inhibited with 10 µg/mL Cap-100. Cellular senescence was assessed by SA-β-Gal staining and flow cytometry; proliferation and apoptosis by CCK-8 and Annexin V/PI, respectively. CAR-T cytotoxicity was evaluated via flow cytometry. CCR7 expression on T cells from B-NHL patients and healthy controls was measured. Lentiviral transduction regulated CCR7 expression; protein expression and interactions were analyzed by Western blot and Co-IP. NF-κB and ARHGAP/RhoA pathways were inhibited using GS143 and Y27632, respectively.

**Results:**

CCR7 overexpression enhanced SASP, while knockdown attenuated it. In the Dox-induced TIS model, KMT2D decreased, whereas H3K9me3, CCR7, and LGALS9 increased; CCR7 inhibition reversed these changes. Cap-100 improved CD19 CAR T killing by alleviating exhaustion in co-culture. Mechanistically, Cap-100 stabilized IκBα to inhibit NFκB, and inhibition of NFκB or ROCK reversed pro-senescence. Cap-100 reduced T cell proliferation but did not affect apoptosis or exhaustion. Clinically, CCR7 was lower on T cells from B NHL patients than healthy controls, and higher CCR7 correlated with better T cell quality. Transcriptomics showed Dox activated TIM3/Galectin 9 and PD- 1/PD-L1 pathways, while Dox + Cap-100 suppressed them. Co-IP confirmed interactions among CCR7, KMT2D, and LGALS9. Collectively, Dox induced TIS to upregulate CCR7, increasing SASP and LGALS9, which interacted with TIM-3, causing immune cell exhaustion and reducing killing efficiency; CCR7 blockade reversed this state and enhanced cytotoxicity.

**CONCLUSIONS:**

Our findings demonstrate that blockade of KMT2D-CCR7-mediated senescence enhances CD19 CAR-T cell anti-tumor activity in B-NHL.

**Graphical abstract:**

**Figure:**
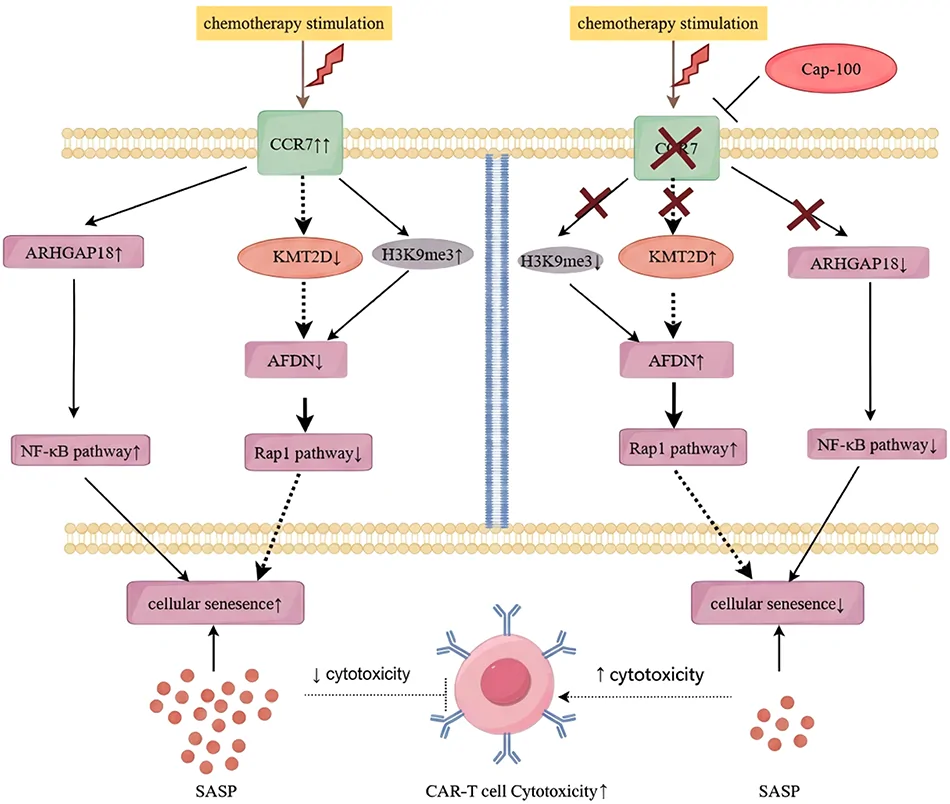
Chemotherapy induces DNA damage, activating NF-κB and ARHGAP18/IKBα pathways, upregulating CCR7, and promoting cellular senescence with increased SASP, which reduces CAR-T cell efficacy. DNA damage also decreases KMT2D and AFDN expression, increases H3K9me3, and inhibits the Rap1 pathway, further sustaining NF-κB activation and CCR7 expression. The CCR7-blocking antibody Cap-100 reverses this by restoring KMT2D and AFDN levels, reactivating Rap1, inhibiting NF-κB, reducing senescence, and enhancing CAR-T-mediated tumor killing

**Supplementary Information:**

The online version contains supplementary material available at https://doi.org/10.1186/s12916-026-05030-4.

## Background

Non-Hodgkin’s lymphoma (NHL) is a hematological malignancy that is characterised by significant cellular heterogeneity, potentially arising from B cells, T cells, or natural killer (NK) cells. B-cell NHL (B-NHL) accounts for approximately 70–80% of all cases [1]. The current standard first-line therapy for B-NHL combines rituximab with the CHOP regimen [2]. However, patients with relapsed or refractory (r/r) disease often respond poorly to existing therapies, with a median survival of just months [3]. Chimeric antigen receptor (CAR) T-cell therapy has emerged as a potent treatment option for r/r B-NHL, demonstrating significant efficacy [4]. Despite the significant efficacy of CD19-targeted CAR-T therapy in B-NHL patients, a major challenge remains in overcoming therapeutic resistance observed in 30–50% of cases [5]. Moreover, approximately 30% of patients demonstrate an absence of response following CAR-T cell infusion, and more than half of the patients who initially achieve remission eventually experience relapse [6]. Thus, overcoming resistance to CAR-T therapy and reducing treatment failure/relapse rates remain urgent priorities. Notably, immunosuppressive tumor microenvironments (TMEs) contribute to CAR-T resistance [7], with treatment-induced senescence (TIS) emerging as a pivotal mechanism driving TME immune suppression [8].

The process of stress-induced cellular senescence is defined as a state of cell cycle arrest triggered by genotoxicity, carcinogenesis, or oxidative stress, and is characterized by elevated expression of Senescence-Associated β-Galactosidase (SA-β-Gal) and H3 lysine 9 trimethylation (H3K9me3) [8, 9]. Contrary to the conventional view that chemotherapy-induced stress-induced senescence of tumor cells is beneficial for inhibiting tumor growth and progression, recent findings suggest that chemotherapy-induced cellular senescence promotes tumor drug resistance and recurrence, and that CAR-T cells targeting senescent cells prolong mouse survival in senescence-inducing drug combinations [10]. Our previous research linked chemotherapy-induced lymphoma senescence to upregulated CCR7, a metastasis-associated chemokine receptor. Chemotherapy induces senescence in lymphoma cells, and B-NHL-TIS cells exhibit CCR7 overexpression coupled with stemness traits, driving drug resistance [11]. However, whether CCR7-targeting in TIS can synergize with lymphoma therapy remains unclear. Studies have shown that senescent cells inhibit T cell activity by secreting IL-10, TGF-β, and other factors [12]. Therefore, this study aims to investigate whether targeting CCR7-mediated B-NHL-TIS can synergistically enhance the efficacy of CAR-T cell therapy and elucidate the underlying mechanisms.

This study investigates how CCR7 blockade reverses TIS-mediated immunosuppression to synergize with CAR-T therapy in B-NHL. Furthermore, we aim to elucidate the mechanism by which CCR7 promotes pro-survival signaling in B-NHL-TIS. By correlating CCR7 expression with clinical CAR-T responses, this work proposes a novel combination strategy targeting CCR7/TIS to overcome therapy resistance. Our findings demonstrate that integrating CCR7 modulation with immunotherapy is a viable approach to improve outcomes in r/r B-NHL.

## Methods

### Patients

We enrolled 111 B-NHL patients and 20 healthy donors in this study from March 2021 to December 2023. Table 1 summarizes the baseline characteristics of the total individuals in this study. The B-NHL cohort included the following clinically defined subtypes: diffuse large B-cell lymphoma (DLBCL, *n* = 25), chronic lymphocytic leukemia (CLL, *n* = 27), mantle cell lymphoma (MCL, *n* = 8), lymphoplasmacytic lymphoma (LPL, *n* = 4), and mucosa-associated lymphoid tissue lymphoma (MALT, *n* = 3). Additionally, 44 patients diagnosed with B-cell non-Hodgkin lymphoma but not assigned to a specific subtype were categorized as “unclassified B-cell non-Hodgkin lymphoma”. The mean age ± standard deviation for the entire cohort was 57.44 ± 17.57 years. Mean age ± standard deviation for each subtype group was as follows: Healthy control group33.9 ± 12.17 years; Diffuse large B-cell lymphoma group: 61.50 ± 12.81 years; Chronic lymphocytic leukemia group: 63.93 ± 9.62 years; Mantle cell lymphoma group: 63.12 ± 7.20 years; Lymphoplasmacytic Lymphoma 50.50 ± 21.36 years; Mucosa-associated lymphoid tissue (MALT) lymphoma group: 71.67 ± 5.77 years; Unclassified B-cell non-Hodgkin lymphoma group: 61.24 ± 16.66 years, there were no significant differences in age or gender among the patient groups. The research study was formally endorsed by the Institutional Review Board of the Second Affiliated Hospital of Anhui Medical University (No. 2021018). All participants in the study provided written documentation confirming their informed consent.

**Table 1:** General clinical baseline data of B-NHL patients

Variables	Total (*n* = 131)		HC (*n* = 20)	DLBC (*n* = 25)	CLL (*n* = 27)	MCL (*n* = 8)	LPL (*n* = 4)	MALT (*n* = 3)	B-NHL (*n* = 44)
Age, Mean ± SD		57.44 ± 17.57	33.90 ± 12.17	61.50 ± 12.81	63.93 ± 9.62	63.12 ± 7.20	50.50 ± 21.36	71.67 ± 5.77	61.24 ± 16.66
Sex, n (%)									
Male		75 (57.25%)	10 (50.00%)	18 (72.00%)	13 (48.15%)	2 (25.00%)	2 (50.00%)	2 (66.67%)	28 (63.64%)
Female		56 (42.75%)	10 (50.00%)	7 (28.00%)	14 (51.85%)	6 (75.00%)	2 (50.00%)	1 (33.33%)	16 (36.36%)

F: ANOVA, -: Fisher’s exact

SD: standard deviation

### Cell culture

The human B-NHL cell lines Raji and SU-DHL-2 were obtained from the China Center for Type Culture Collection and cultivated in RPMI-1640 (Hyclone, Logan, Utah, USA; SH30809.01) containing 10% foetal bovine serum (Lonza, Uruguay; S711-001 S). The cultivation temperature was maintained at 37 °C, and the environment was in a humidified incubator with 5% CO_2_. Additionally, regular PCR testing was performed to confirm the absence of mycoplasma contamination.

### Lentiviral transfection

To modulate CCR7 expression, cells were transduced with a recombinant lentivirus targeting CCR7 (CCR7i) for knockdown or with a CCR7-overexpressing lentivirus (CCR7). Corresponding empty vectors (Vector1 for CCR7i, Vector2 for CCR7) were utilized as negative controls. All lentiviruses were procured from GeneChem (Shanghai, China). The cells were then exposed to lentivirus for a period of 48 h, after which puromycin selection was performed. The expression levels of CCR7 were confirmed by quantitative reverse transcription PCR (qRT-PCR) and western blot analysis.

### Doxorubicin, GS143 and Y-27,632 preparation

Doxorubicin hydrochloride (≥ 98.0%, ST1285, Beyotime Biotechnology, China) was dissolved in 1.72 mL of DMSO to prepare a 10 mM stock solution. GS143 (HY-110261) and Y-27,632 (HY-10071) were purchased from MedChem Express (MCE, New Jersey, USA) and prepared in accordance with the manufacturer’s instructions. The final working dilutions of 0.1, 1, 10, and 100 µM were obtained by appropriate dilution of the stocks in culture medium before treatment.

### Enzyme-linked immunosorbent assay (ELISA)

Cell culture supernatants and patient serum samples were collected and processed according to the instructions provided by the commercial ELISA kits. All reagents were equilibrated to room temperature before use. Three sets of wells were prepared: blanks (sample diluent only), standards, and sample wells (cell culture supernatant or serum). Each well was then incubated with 100 µl of HRP-conjugated detection antibody at 37 °C for 60 min in the dark. After incubation, 100 µl of substrate solution (AB mix) was added and further incubated for 15 min in the dark. The reaction was stopped by adding 50 µL of stop solution, and absorbance was measured at 450 nm. Standard curves were generated using OD values from the standards, and only curves with an R² value > 0.99 were accepted. The OD readings showed a positive relationship with analyte concentrations. Multiple commercial ELISA kits (RUIXINBIO, China) were used to determine cytokine and surface marker levels in human samples. Detailed kit information, including targets, catalog numbers, and manufacturers, is provided in Additional file 1: Table 2.

### Multiplex bead-based flow cytometric immunoassay for cytokine measurement

The quantification of cytokines in the Raji cell co-culture medium was accomplished using a multiplex bead-based flow cytometric immunoassay with 12 distinct cytokine testing kits manufactured by Qingdao RaiseCare Biological Technology Co. (Registration No.: LUMAOZIQUAN 20222400659; Production Batch No: 20250202). The test was conducted in accordance with the kit instructions.

### Flow cytometry

### Cell senescence detection

Cells cultured under optimal conditions were treated with Dox and Cap-100 (research-grade anti-human CD197/CCR7, Biotech Co. Ltd., China) and harvested at 0, 24, 48, and 72 h after treatment. Cells were first stained with CD19 antibody, then fixed and permeabilized. After blocking, cells were incubated with primary antibody against H3K9me3 (tri-methyl K9, ab88998, Abcam) for 60 min, followed by centrifugation and removal of supernatant. Subsequently, cells were incubated with a pre-adsorbed secondary antibody (Alexa Fluor® 488, ab150081, Abcam) for 30 min in the dark. Cell populations were identified by forward scatter (FSC) and side scatter (SSC), and lymphoma cells were gated as CD19⁺. The presence of H3K9me3 within the nucleus of CD19⁺ cells was assessed as a marker of cellular senescence. The monoclonal antibodies used in this study were purchased from Beckman Coulter Immunology (Miami, USA) and BioLegend (San Diego, USA). Samples were analyzed using a CytoFLEX flow cytometer (Beckman Coulter, USA).

### Apoptosis detection

The process of apoptosis was detected using the Annexin V-APC/PI double staining method (BestBio, Shanghai, China). The cell concentration was adjusted to 1 × 10^5^ cells/mL, and the cells were washed three times using phosphate buffer solution (PBS). Propidium iodide (PI) and Annexin V-APC were then added, and the mixture was incubated for 15 min at room temperature. The samples were then analysed using flow cytometry (BD Biosciences).

### Cell counting kit-8 (CCK-8) assay

Raji and SU-DHL-2 cells were seeded into 96-well plates and treated with Dox and Cap-100 (a functional blocking antibody against CCR7) according to the protocol. Then, 100 µl of medium containing 10 µl of CCK-8 reagent (MedChem Express, USA, HY-K0301) was added to each well. After 2 h of incubation, the supernatant was collected, and the absorbance was measured at 450 nm using a microplate reader. Cell viability was calculated using the following formula: (Aₛ - A_b) / (A_c - A_b) × 100%, where Aₛ is the absorbance value of the experimental group (cells + medium + CCK-8 + drug), A_c is the absorbance value of the control group (medium + CCK-8 + cells, no drug), and A_b is the absorbance value of the blank group (medium + CCK-8 only, no cells or drug). All experiments were repeated three times independently, with five parallel wells in each group.

### Soft agar colony formation assay

Following a 72-hour treatment period across the four groups previously delineated, Raji and SU-DHL-2 cells were counted. Cells were then resuspended in fresh medium and seeded at a density of 1 × 10^3^ cells per well in 12-well plates. Cultures were maintained in complete medium containing 10% foetal bovine serum (FBS) at 37 °C and 5% carbon dioxide (CO₂) for 10–14 days. Following culture, the cells were stained with crystal violet for 2 h, after which colony counts were determined under a light microscope. The calculation of plating efficiency (PE) and surviving fraction (SF) was conducted using the following formulae (Colony formation rate = Number of colonies / Number of inoculated cells × 100%). Each group comprised triplicates, and the experiment was repeated a minimum of three times. The data are presented as the mean ± standard deviation. Statistical analysis was performed using Student’s t-test or one-way ANOVA (*p* < 0.05 considered significant).

### RNA-sequencing

In this study, RNA was extracted from Raji cells after 72 h of treatment (Con, Dox, Cap-100, Dox + Cap-100). Total RNA was extracted using the TRIzol method, and RNA concentration and purity were subsequently measured. The RNA samples were then sent to BioMarker for high-throughput sequencing of the transcriptome (Illumina NovaSeq 6000 platform, PE150). The raw data underwent a series of quality control and filtration steps using the fastp tool. A comparison of the data was conducted using the HISAT2 method, followed by quantification of gene expression based on the featureCounts tool. The identification of differentially expressed genes (DEGs) was performed using the DESeq2 software (|log₂FC| > 1.5, *p* < 0.05). Subsequently, differential genes were analyzed for Gene Ontology (GO) and Kyoto Encyclopedia of Genes and Genomes (KEGG) enrichment, and significant pathways were screened at a threshold of *p* < 0.05.

### Western blotting

The proteins were separated on 4–12% Criterion XT Bis-Tris gels (Bio-Rad) after the cells were lysed with RIPA lysis buffer (Thermo Fisher Scientific, #89900) and transferred to PVDF membranes (Bio-Rad). The membranes were blocked with 5% skimmed milk powder at room temperature for 2 h, then incubated with the primary antibody at 4 °C overnight. The membranes were then washed with TBST, incubated with the HRP-conjugated secondary antibody (Promega, 1:5000) at room temperature for 1 h, and then washed three times. The bands were detected using enhanced chemiluminescence (Thermo Fisher Scientific), and the images were acquired using an Eagle Chemi Analyzer (Beijing Baijing). The primary antibodies are listed in Additional file 1:Table 3 and were diluted with either 5% skimmed milk powder or BSA, both of which are validated and commonly used.

### Co-immunoprecipitation (Co-IP)

The cells were washed with pre-chilled PBS, and the cell lysate was prepared (RIPA: PMSF = 100:1). Protein A + G magnetic beads (BeyoMagTM protein A + G Magnetic Beads, P2102-5 ml, China) were used according to the manufacturer’s instructions. The cell lysates were divided into the IgG and IP groups (IgG-CCR7, IP-CCR7, IgG-KMT2D, IP-KMT2D, IgG-LGALS9, IP-LGALS9). The final concentration of antibodies was 500 µg/ml Incubated in a shaker at 4℃ for 6 h. The beads were washed 3 times with RIPA buffer using a magnetic rack. The magnetic beads were resuspended in 50 µL of RIPA buffer, mixed with loading buffer, and then boiled at 100 °C for 10 min. After denaturation, the samples were centrifuged at 12,000×g for 2 min, and the supernatants were analyzed by Western Blotting.

### In vivo antitumor effect of CAR-T cells

The NSG mice required for the in vivo experiments were purchased from Cyagen (Suzhou) Biotechnology Co., Ltd. In accordance with the standard operating procedures for animal welfare established by the Institute of Health Sciences at the Hefei Comprehensive National Science Center, 6-week-old female NSG mice (*n* = 5) were injected with 1 × 10⁶ treated Raji-Luciferase cells into the right axilla. On the seventh day following the injection of tumor cells, mice in the different treatment groups were randomly divided into three groups and treated via tail vein injection with PBS or 4 × 10⁶ Mock-T/CAR-T cells, respectively. Blood samples (200 µl) were collected from the cheeks of the mice on days 3, 14, and 28 after T-cell infusion, and flow cytometry was used to detect residual and exhausted T cells. Mouse body weight was measured every 3 days, and in vivo tumor imaging was performed every 7 days using the PerkinElmer IVIS X5 system. This involved intraperitoneal injection of 150 mg/kg D-Luciferin potassium salt (MedChem Express, HY-125918); after a 5-minute wait, the mice were anesthetized with isoflurane and imaged. This procedure began on the 7th day after tumor cell injection and was performed once weekly until the end of the experiment; images were analyzed using Living Image, V.4.1, software (PerkinElmer).

### CAR-T killing assay

Tumor cells were subjected to a 48-hour pre-treatment with Cap-100, followed by co-culture with CD19 CAR-T cells or Mock-transduced T cells (Mock-T cells) at an effector-to-target (E: T) ratio of 1:1 (1 × 10^5^:1 × 10^5^). At 0 and 24 h post-coculture, cells were stained with PC7-labeled CD45 (clone J33), FITC-labeled CD3 (clone A08932), and PE-labeled CD19 (clone A07769), followed by analysis using flow cytometry (CytoFLEX, Beckman Coulter, USA).

### Statistical analysis

All the statistical analyses were conducted using two software programmes: Microsoft Excel and GraphPad Prism version 10.1.2 (GraphPad Software Inc.). For clinical characteristics, differences in sex ratio were analysed using Fisher’s exact test, and age differences were assessed using one-way analysis of variance (ANOVA). The normality of continuous variables was examined using the ShapiroWilk test. Data are presented as mean ± standard deviation (SD). For normally distributed data, parametric tests were applied; non-parametric tests were used when the data did not meet normality assumptions. Correlations between variables were evaluated using Spearman’s rank correlation analysis. Survival outcomes were assessed using KaplanMeier curves, and group differences were compared using the log-rank test. *p* < 0.05 was considered statistically significant.

## Results

### Blockade of CCR7 reduces B-NHL-TIS and enhances tumor cell apoptosis

CCR7 was knocked down (Sh2) or overexpressed (Over) in Raji cells using lentiviral transduction. Cells were treated with or without Dox, and culture supernatants were collected for ELISA to assess cytokine secretion (Additional file 2:S1). Dox significantly increased CCR7 expression in the NC, Vec, and especially the Over groups, but not in the Sh2 group, confirming effective modulation of CCR7. Dox also elevated CCL21 levels in the Sh2 and Vec groups, but not further in the Over group, suggesting potential negative feedback. Stemness and migration markers CD44 and CD34 were significantly upregulated in the Sh2 and Vec groups under Dox, but unchanged in the Over group, implying that CCR7 knockdown promotes CD44/CD34 expression. The inflammatory regulator IκBα was upregulated only in the NC group after Dox, indicating that its increase depends on normal CCR7 expression. LGR5 expression rose significantly in the NC, Vec, and Over groups but remained unchanged in the Sh2 group, suggesting that CCR7 supports LGR5 expression. Overall, these results indicate that CCR7 modulates inflammatory suppression, stemness, and migration in B lymphoma by regulating CCL21, CD44, CD34, IκBα, and LGR5 in a dose-dependent manner, highlighting its role in cell fate and microenvironmental adaptation.

**Fig. 1:**
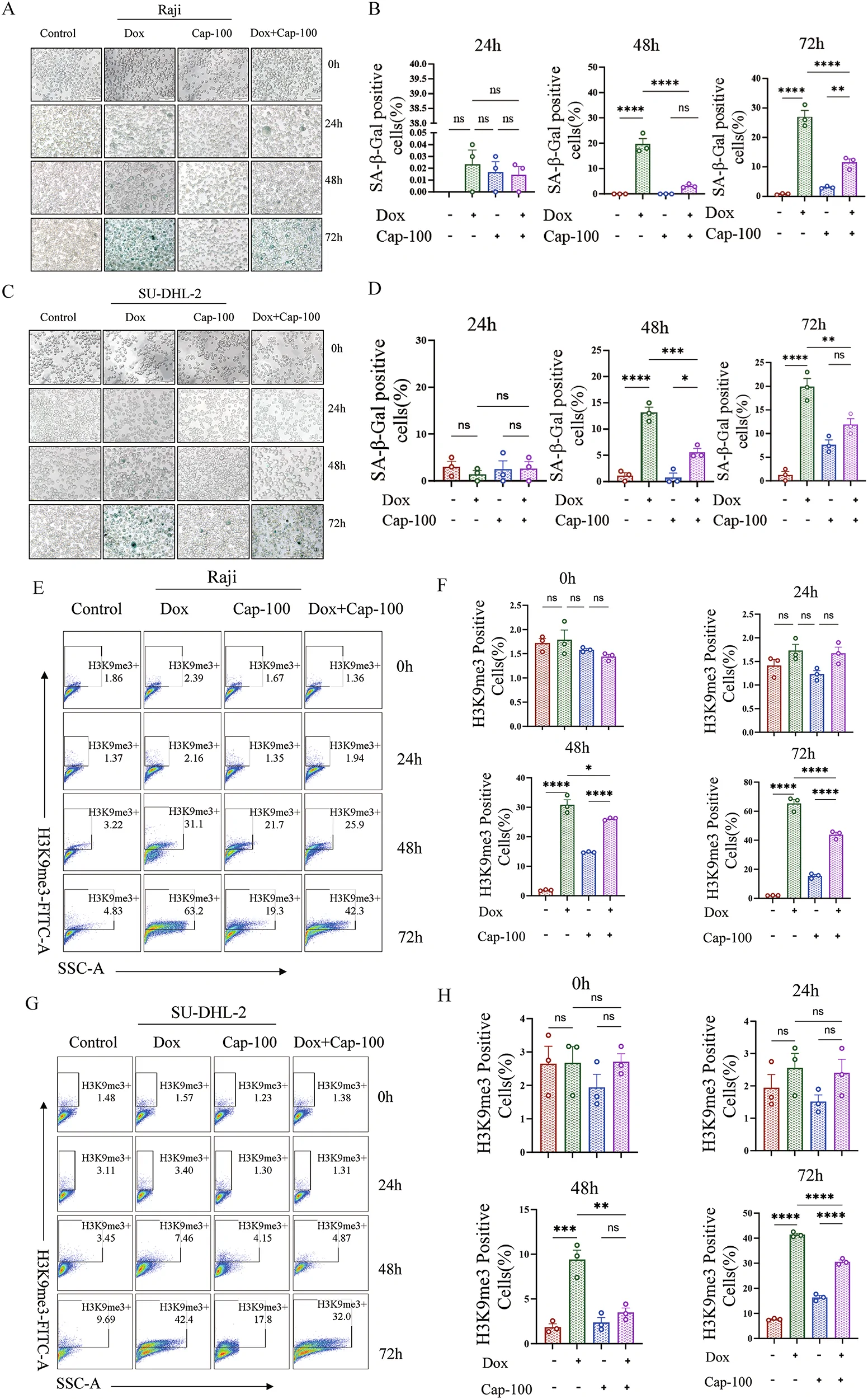
Blockade of CCR7 Reduces B-NHL-TIS and Enhances Tumor Cell Apoptosis. **A** Senescence staining was used to detect changes in SA-β-Gal in Raji cells at different time points under different treatment conditions. **B** Statistical graph of senescence staining in Raji cells. **C** Senescence staining was used to detect changes in SA-β-Gal in SU-DHL-2 cells at different time points under different treatment conditions. **D** Statistical graph of senescence staining of SU-DHL-2 cells. **E** Flow cytometry to detect the proportion of H3K9me3 in Raji cells at different time points under different treatment conditions. **F** Statistical graph of the proportion of H3K9me3 in Raji cells detected by flow cytometry. **G** Proportion of H3K9me3 in SU-DHL-2 cells at different time points under different treatment conditions by flow cytometry. **H** Statistical graph of the percentage of H3K9me3 in SU-DHL-2 cells detected by flow cytometry. All results were repeated at least three times. *p < 0.05, **p < 0.01, ***p < 0.001, ****p < 0.0001

**Fig. 2:**
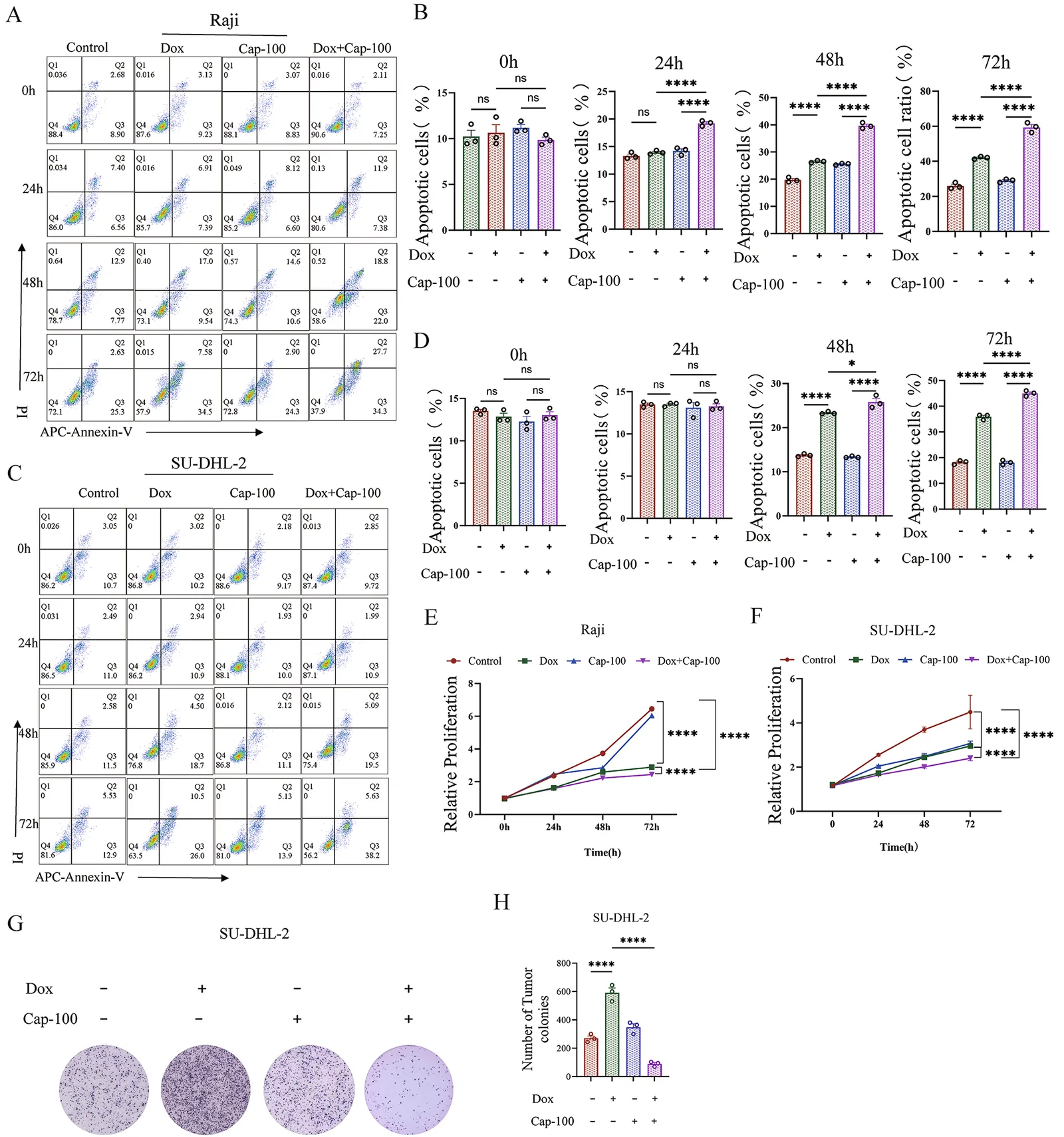
Combined action of Dox and Cap-100 increases apoptosis in B-NHL cells. **A** Percentage of apoptosis of Raji cells detected by flow cytometry at different time points under different treatment conditions. **B** A statistical graph of apoptosis of Raji cells was detected by flow cytometry at different time points under different treatment conditions. **C** The percentage of apoptosis of SU-DHL-2 cells was detected by flow cytometry at different time points under different treatment conditions. **D** Statistical graph of apoptosis of SU-DHL-2 cells detected by flow cytometry at different time points under different treatment conditions. **E** Cell proliferation of Raji cells was detected by CCK8 at different time points under different treatment conditions. **F** Cell proliferation of SU-DHL-2 cells was detected with CCK8 under different treatment conditions. **G** The colony-forming assay assesses the long-term proliferative capacity of SU-DHL cells treated under identical conditions.H SU-DHL-2 Cell Proliferation and Colony Count Statistical Chart. All results were repeated at least three times. *p < 0.05, **p < 0.01, ***p < 0.001, ****p < 0.0001

We hypothesized that CCR7 regulates cytokine secretion, cellular senescence, apoptosis, and proliferation. To further investigate these observations, Raji and SU-DHL-2 cells were treated with Dox alone or in combination with the CCR7-blocking antibody Cap-100. Flow cytometry was performed at multiple time points to assess changes in CCR7 expression on the cell surface under different treatment conditions. The results showed that Dox treatment led to an upregulation of membrane CCR7, whereas the addition of Cap-100 attenuated this increase and resulted in a reduction of CCR7 expression(Additional file 2:S2A, S2B, S2E, S2F). Senescence was assessed by SA-β-Gal staining and H3K9me3 flow cytometry. Apoptosis was measured by Annexin V-PI assay, and proliferation by CCK-8 assay. The results demonstrated that Dox treatment led to a significant increase in senescent cell proportions at 48 and 72 h. In contrast, the combination of Dox and Cap-100 resulted in a reduction of senescence when compared to Dox treatment alone (Fig. 1A–D), and levels of H3K9me3 exhibited a congruent response to these observations (Fig. 1E-H). The process of apoptosis was augmented by Dox alone, with a further increase in the Dox + Cap-100 group (Fig. 2A–D). The proliferation of these cells was effectively suppressed by both treatments, with the Dox + Cap-100 group demonstrating a significantly more pronounced suppression compared to Dox alone (Fig. 2E, F). The reliability of this result was indirectly corroborated by measuring the changes in average cell diameter and cell growth density across four subgroups of cells from both cell lines. Compared with the Dox group, the Cap-100 group exhibited both a smaller mean cell diameter and lower cell density. (Additional file 2:S2C, S2D, S2G, and S2H).Cap-100 treatment alone had no significant effect on colony formation compared to the untreated control. Cells that survived the initial Dox-induced senescence stimulus exhibited a marked increase in clonogenic capacity. This suggests that the surviving subpopulation possesses enhanced proliferative potential. Most critically, this pro-colony formation was significantly attenuated in the Dox + Cap-100 combination group. The number and size of colonies in this group were substantially reduced compared to the Cap-100 alone group. Our findings demonstrate that concurrent inhibition of CCR7 signaling via Cap-100 can counteract this undesirable effect by suppressing the clonogenic expansion of senescence-surviving cells. In summary, Dox induces senescence, apoptosis, and CCR7 expression, and inhibits proliferation in B-NHL cells, while CCR7 blockade reduces Dox-induced senescence, enhances apoptosis, and further suppresses proliferation, highlighting CCR7’s key role in regulating B lymphoma cell fate.

### Blockade of CCR7 shows minimal direct effects on T-cell apoptosis or exhaustion but partially suppresses proliferation

**Fig. 3:**
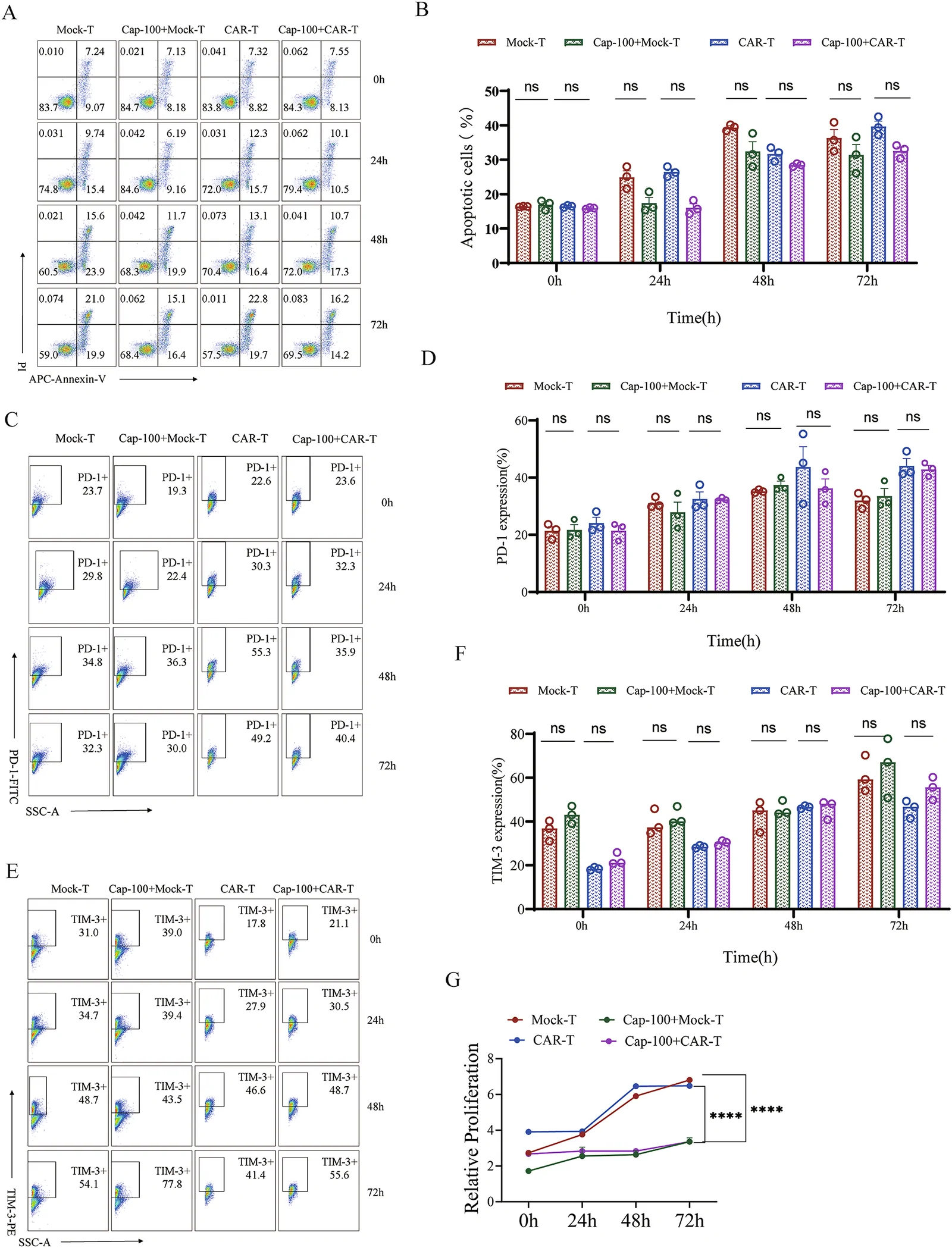
Alterations in T-cell apoptosis, exhaustion, and proliferation following CCR7 blockade. **A** Percentage of apoptosis of Mock-T/CAR-T cells detected by flow cytometry at different time points under different treatment conditions. **B** A statistical graph of apoptosis of Mock-T/CAR-T cells was detected by flow cytometry at different time points under different treatment conditions. **C** The percentage of PD-1 on Mock-T/CAR-T cells was detected by flow cytometry at different time points under different treatment conditions. **D** Statistical graph of PD-1 on Mock-T/CAR-T cells detected by flow cytometry at different time points under different treatment conditions. **E** The percentage of TIM-3 Mock-T/CAR-T cells was detected by flow cytometry at different time points under different treatment conditions. **F** Statistical graph of TIM-3 of Mock-T/CAR-T cells detected by flow cytometry at different time points under different treatment conditions. **G** Cell proliferation of Mock-T/CAR-T cells was detected by CCK8 at different time points under different treatment conditions. All results were repeated at least three times. *p < 0.05, **p < 0.01, ***p < 0.001, ****p < 0.0001

To exclude the possibility that Cap-100 directly affects T cells and thereby influences CAR-T killing outcomes, we evaluated whether Cap-100 has direct effects on CAR-T or Mock-T cells. In addition to the experiments focused on tumour cells, we evaluated whether Cap-100 has direct effects on CAR-T or Mock-T cells. Cap-100 was added to the T cells, and apoptosis and exhaustion markers were examined using flow cytometry at 0, 24, 48, and 72 h. Cell proliferation was assessed using the CCK-8 assay. The results showed that Cap-100 treatment did not lead to any noticeable changes in apoptosis or exhaustion in either CAR-T or Mock-T cells (Fig. 3A–F). This suggests that blocking CCR7 does not cause harmful activation or survival defects in T cells.

However, Cap-100 treatment resulted in a significant decrease in the proliferative capacity of both CAR-T and Mock-T cells (Fig. 3G). These findings suggest that, while CCR7 inhibition may restrict T-cell expansion, it does not cause T-cell exhaustion or apoptosis. This T-cell-intrinsic effect appears distinct from the pro-apoptotic and anti-senescence roles that Cap-100 exerts in Dox-induced senescent lymphoma cells.

### Clinical relevance of CD19-CAR-T treatment-related cytokine changes and T-cell CCR7 expression

To validate the clinical relevance of the above in vitro findings, we monitored the dynamics of tumor-related factors during CD19-CAR-T therapy in patients with B-ALL(Additional file 2:S3). Plasma samples were collected at specific intervals and grouped based on clinical response: progressive disease (PD), new diagnosis (ND), and relapse/refractory (r/r). Multiplex cytokine analysis revealed distinct patterns among the groups (Additional file 2:S2). On the seventh day, IκBα, a pivotal inflammatory modulator, exhibited a substantial increase in the ND group, while it demonstrated a decrease in the PD and r/r groups. This finding suggests that IκBα contributes to early immune regulation, and its decrease in PD or r/r patients may reflect insufficient immunosuppression. TGF-β1 levels remained elevated in PD, suggesting the presence of an immunosuppressive microenvironment associated with therapy resistance. The chemokines CCL21 and CCL19 exhibited increased expression in r/r but decreased expression in ND, suggesting their involvement in the tumor microenvironment and cell migration. IL-35 demonstrated a transient increase in ND, yet remained elevated in PD. Concurrently, SLAMF1 expression was augmented in ND, suggesting a potential enhancement in immune clearance mechanisms. These dynamic changes correlate with treatment outcomes, highlighting CCR7 pathway-related factors (IκBα, CCL21, CD44) in regulating the tumor microenvironment and CAR-T efficacy.

**Fig. 4:**
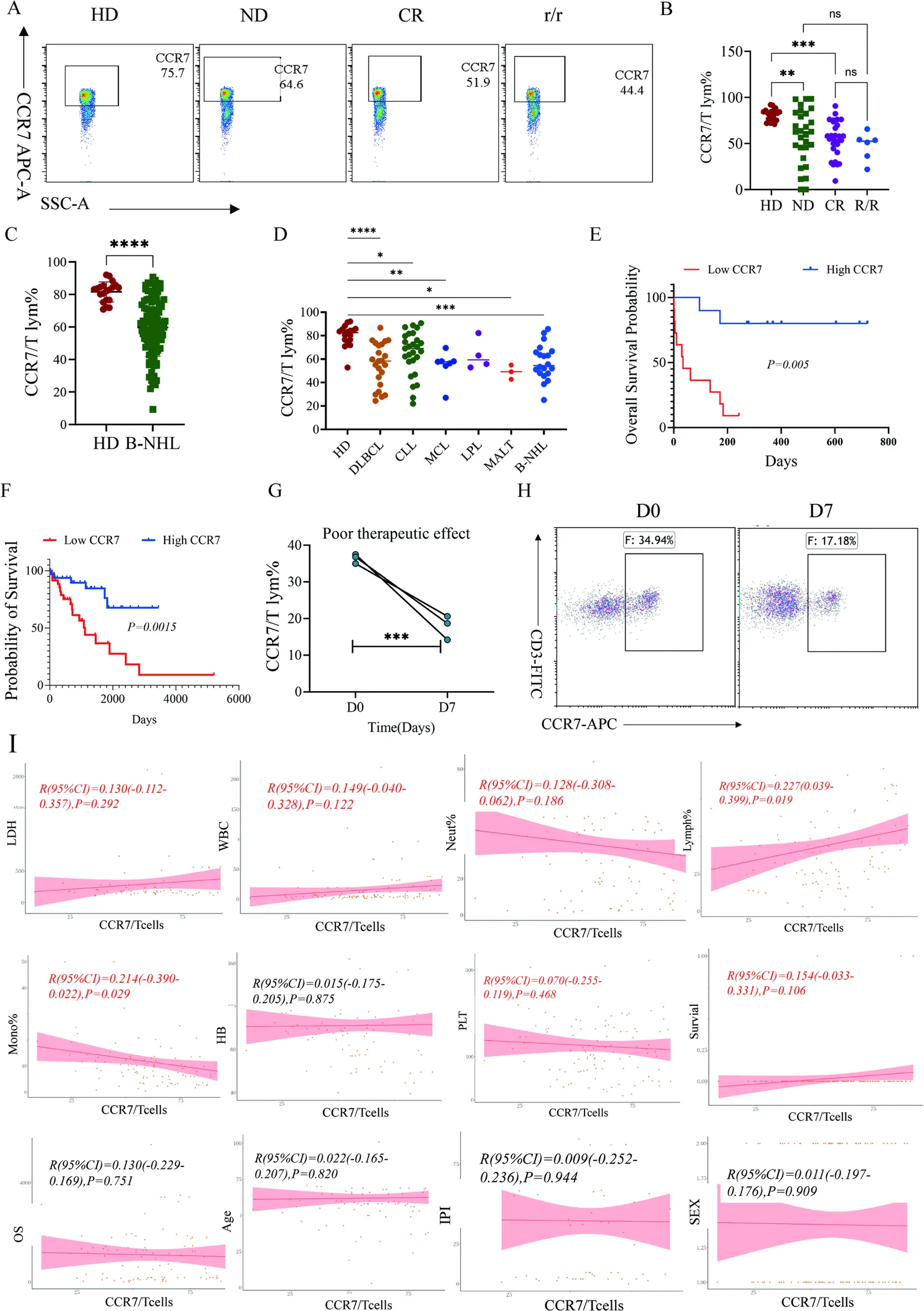
Correlation Analysis of CCR7 Expression in T Cells of B-NHL Patients. **A** Representative flow plots of CCR7 expression in T cells of B-NHL patients with different disease states. **B** All enrolled patients were divided into four groups: healthy controls (HD), newly diagnosed (ND), in complete remission (CR), and relapsed/refractory (R/R). These groups were then statistically analyzed. **C** All enrolled individuals were divided into the HD group and the patient group, and were statistically analyzed. **D** Patients were categorized by lymphoma subtype, and CCR7 expression on T cells was statistically analyzed. **E** The critical value was used as the cutoff point to categorize patients as having high or low expression. Kaplan-Meier (KM) survival curves were plotted. **F** Kaplan–Meier curves for progression-free survival (PFS) according to CCR7 expression on T cells in patients with B-NHL. **G** CCR7 expression on T cells in DLBCL patients receiving CAR-T therapy. **G** Changes in CCR7 expression on T cells in patients with diffuse large B-cell lymphoma receiving CAR-T therapy. **H** Representative figure showing CCR7 expression on T cells from patients with diffuse large B-cell lymphoma undergoing CAR-T therapy. **I** Correlations between CCR7 expression on T cells and high-risk factors for lymphoma, such as age, LDH, ECOG scores, IPI scores, percentages of WBC, lymphocytes, monocytes, neutrophils, PLT, and HB were examined. *p < 0.05, **p < 0.01, ***p < 0.001, ****p < 0.0001

Preliminary studies by our team indicate that, among patients with relapsed/refractory B-cell non-Hodgkin lymphoma, lymphoma cells exhibit the highest levels of CCR7 expression on their surface, appearing strongly positive. CCR7 is an important chemokine receptor that plays a key role in T cell homing and function. It is found in both CAR-T cells and endogenous T cells from B-NHL patients. Subsequently, we assessed CCR7 expression levels in peripheral blood T cells from both B-cell non-Hodgkin lymphoma patients and healthy donors (Fig. 4A). Results revealed markedly diminished CCR7 expression in B-NHL patients, particularly within the relapsed/refractory (R/R) cohort and across distinct lymphoma subtypes (Fig. 4B–D). Kaplan–Meier survival curve analysis revealed that patients with high CCR7 expression (≥ 62.8) had a significantly higher overall survival rate at 200 days (log-rank *P* = 0.005) (Fig. 4E). The progression-free survival (PFS) differed significantly between the high- and low-CCR7 groups (cut at median CCR7 expression). The median PFS was not reached in the high-CCR7 group, whereas it was 1115 days in the low-CCR7 group. The log-rank test demonstrated a statistically significant difference in PFS (χ² = 10.06, *P* = 0.0015, Fig. 4F). The hazard ratio (HR) for progression in the high-CCR7 group compared with the low-CCR7 group was 0.255 (95% CI: 0.1138–0.5720), indicating a substantially lower risk of progression in patients with high CCR7 expression. Monitoring of T-cell CCR7 expression in three B-NHL patients with poor response to CAR-T therapy revealed a consistent downward trend in CCR7 expression on T cells from Day 0 (D0) to Day 7 (D7) post-CAR-T treatment. Efficacy assessments demonstrated that the dynamic changes in CCR7 following CAR-T infusion were closely correlated with treatment outcomes (Fig. 4G, H). This pattern, where lymphoma cells exhibit the highest CCR7 expression and T cells the lowest in R/R B-NHL patients, suggests tumour-immune imbalance: high CCR7 expression in tumour cells may enhance lymph node homing, survival, and stemness, while low CCR7 expression in T cells may impair lymphoid tissue migration and induce T cell exhaustion. Collectively, these features promote immune evasion, providing a mechanistic basis for the impact of treatment-induced senescence on tumour progression. Correlation analysis (Fig. 4I) revealed that CCR7 expression was associated with an increased percentage of lymphocytes, but decreased numbers of neutrophils and monocytes. This indicates that CCR7 expression may be correlated with immune cell composition. However, there was no significant correlation with age LDH, ECOG, IPI, WBC, PLT, or HB.The present study demonstrates that CCR7 and its associated signaling axis shape both the tumor microenvironment and T-cell functionality, influence the dynamic response to CAR-T therapy, and serve as a strong predictor of clinical outcomes in B-cell malignancies.

### Pre-treatment with the CCR7-blocking antibody Cap-100 enhances CD19-CAR-T cell-mediated cytotoxicity against B-cell lymphoma cells

**Fig. 5:**
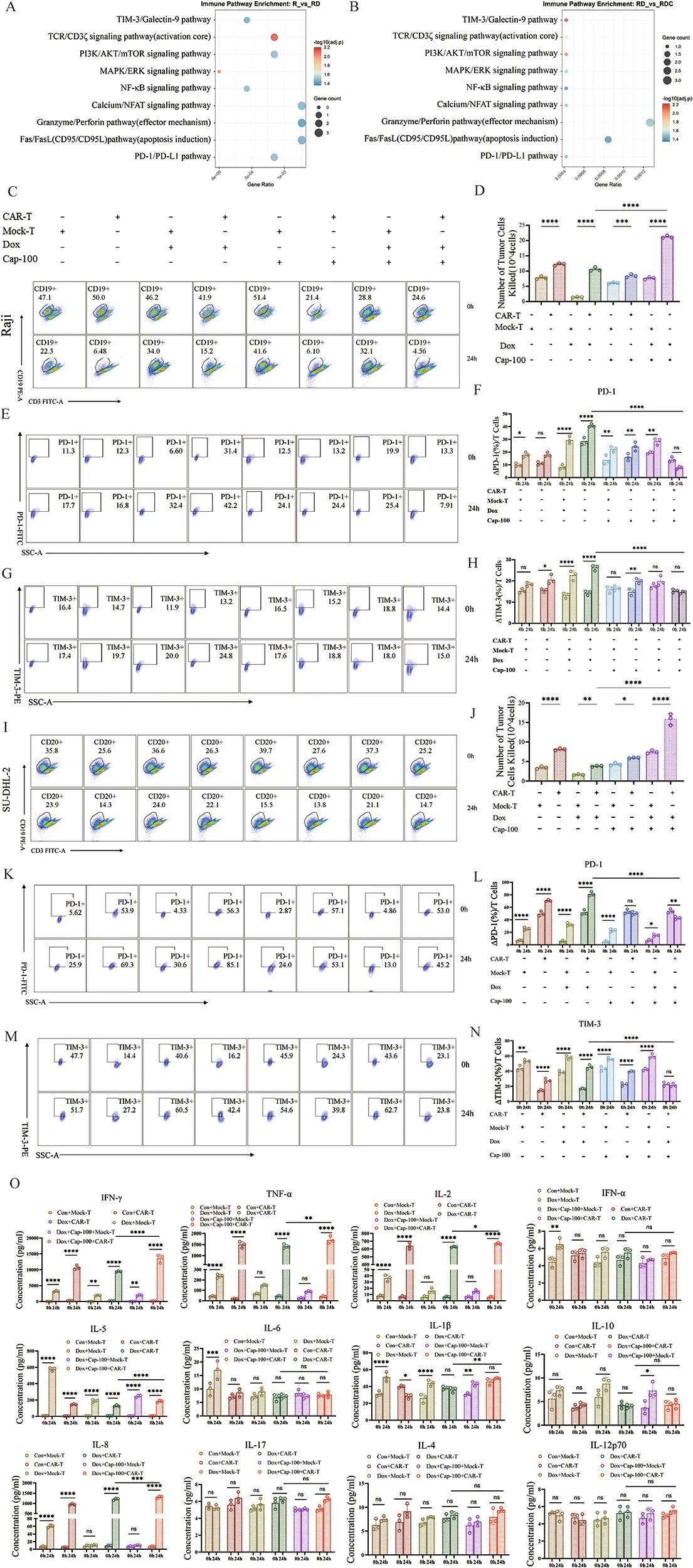
Pre-treatment with the CCR7-blocking antibody Cap-100 enhances CD19-CAR-T cell-mediated cytotoxicity against B-cell lymphoma cells. **A** Immune pathway enrichment bubble plot for differentially expressed genes in Raji cells (Con vs Dox). **B** Immune pathway enrichment bubble plot for differentially expressed genes in Raji cells (Dox vs Dox+Cap-100). **C** Raji cells were co-cultured with Mock-T and CAR-T cells after the same treatment as above for flow-through plots, respectively. **D** A plot of Raji cells was co-cultured with Mock-T cells and CAR-T cells, respectively, after the same treatment as above, and then statistically analyzed according to the reduced number of tumour cells. **E** Flow cytometry analysis of PD-1 expression on T cells across different groups during Raji cells co-culture. **F** Statistical graph of PD-1 expression on T cells across different groups during Raji cells co-culture. **G** Flow cytometry analysis of TIM-3 expression on T cells across different groups during Raji cells co-culture. **H** Statistical graph of TIM-3 expression on T cells across different groups during Raji cells co-culture. **I** Flow chart of SU-DHL-2 cells co-cultured with Mock-T cells and CAR-T cells, respectively, after the same treatment as above. **J** Plot of SU-DHL-2 cells after co-culture with Mock-T cells and CAR-T cells, respectively, according to the same treatment as above, after statistical analysis according to the reduced number of tumour cells. **K** Flow cytometry analysis of PD-1 expression on T cells across different groups during SU-DHL-2 cells co-culture. **L** Statistical graph of PD-1 expression on T cells across different groups during SU-DHL-2 cells co-culture. **M** Flow cytometry analysis of TIM-3 expression on T cells across different groups during SU-DHL-2 cells co-culture. **N** Statistical graph of TIM-3 expression on T cells across different groups during SU-DHL-2 cells co-culture. **O** Changes in IFN-γ.TNF-α, IL-2, IL-8, IL-5, IL-6, IL-1β, IL-10, IFN-α, IL-17, IL-4, IL-12p70 content after co-culture of Raji cells with CAR-T (R). cells. *p < 0.05, **p < 0.01, ***p < 0.001, ****p < 0.0001

Subsequent analysis of clinical samples showed a substantial reduction in CCR7 expression levels in the peripheral T cells of B-NHL patients, especially in the relapsed/refractory group. These results imply that CCR7 could be pivotal in regulating anti-tumour immunity. Based on this, we performed transcriptome sequencing on four groups of Raji cells under the same treatment conditions. The results showed that Dox treatment significantly elevated the expression of the senescence-associated secretory phenotype (SASP) and the senescence-related pathway. However, expression of this pathway decreased upon CCR7 blockade (Fig. 5A–B), consistent with prior experiments. It is noteworthy that exhaustion-related pathways (TIM-3/Galectin-9 Pathway, PD-1/PD-L1 Pathway) exhibited alterations consistent with ageing-related pathways, namely activation in the Dox group and suppression in the Dox + Cap-100 group. It is therefore hypothesised that the suboptimal response to CAR-T therapy in B-NHL patients experiencing disease recurrence after multiple chemotherapy cycles may be associated with chemotherapy-induced immunosuppression, thereby engendering a microenvironment conducive to T-cell exhaustion. In light of the aforementioned findings, a simulation of the clinical scenario was conducted through the following experiment: B-NHL cells (Raji and SU-DHL-2) were treated with Dox and Cap-100 according to the aforementioned groups for 48 h. Following medium exchange, the cells were co-cultured with CAR-T cells to assess the impact of CCR7 functional inactivation on CAR-T cell killing sensitivity. The quantification of tumour cell killing was conducted via flow cytometry and cell counting following a 24-hour co-culture period. Concurrent labelling of exhaustion markers PD-1 and TIM-3 revealed T-cell exhaustion, thereby elucidating the mechanism by which CCR7 modulates CAR-T cell killing efficacy.

The results indicate that after 24 h of co-culture, the CAR-T group demonstrated a significantly higher level of cell killing compared to the Mock-T group, thereby confirming the CAR-T cells’ capacity for cell killing. The experimental findings demonstrated that, in comparison with the Dox group, the Dox + Cap-100 group exhibited a considerably diminished proportion of surviving tumor cells when co-cultured with CAR-T cells, in addition to a higher total number of eliminated tumor cells compared to the Dox group (*p* < 0.01, Fig. 5C and D). A comparable trend was observed in SU-DHL-2 cells (*p* < 0.05, Fig. 5I and J). The depletion assays of T cells in the co-culture system yielded results indicating heightened expression of T cell depletion phenotypes in the Dox group. Conversely, the Dox + Cap-100 group exhibited a substantial reduction in depletion phenotypes (Fig. 5E, H and K–O). The results demonstrate that the inhibition of CCR7 not only reduces TIS formation in lymphoma cells and enhances their susceptibility to CAR-T-mediated toxicity, but also mitigates the induction of T-cell exhaustion during tumour-T-cell interaction. In co-culture assays of CAR-T cells with Raji cells, cytokine levels in the cell culture medium were measured to assess the functional activation of CAR-T cells. The results demonstrated that the levels of IFN-γ, TNF-α, IL-1β, IL-2, IL-5, and IL-8 were significantly higher following co-culture, indicating robust CAR-T activation and cytotoxic function. However, levels of IFN-α, IL-4, IL-6, IL-10, IL-17, and IL-12p70 did not change significantly, suggesting that these cytokines were not substantially involved in the observed immune response under the tested conditions (Fig. 5O).

Overall, our clinical analyses, transcriptomic profiling, and functional assays demonstrate that CCR7 is a central regulator of chemotherapy-induced senescence and tumourimmune interactions in B-NHL. CCR7 blockade not only suppresses senescence- and exhaustion-related pathways but also enhances tumour susceptibility to CAR-T cytotoxicity while limiting T-cell exhaustion. These findings identify CCR7 as a key determinant of CAR-T efficacy and a promising therapeutic target to improve treatment response.

### Blockade of CCR7 enhances CAR-T anti-tumor efficacy and reduces B-NHL-TIS in vivo experiments

**Fig. 6:**
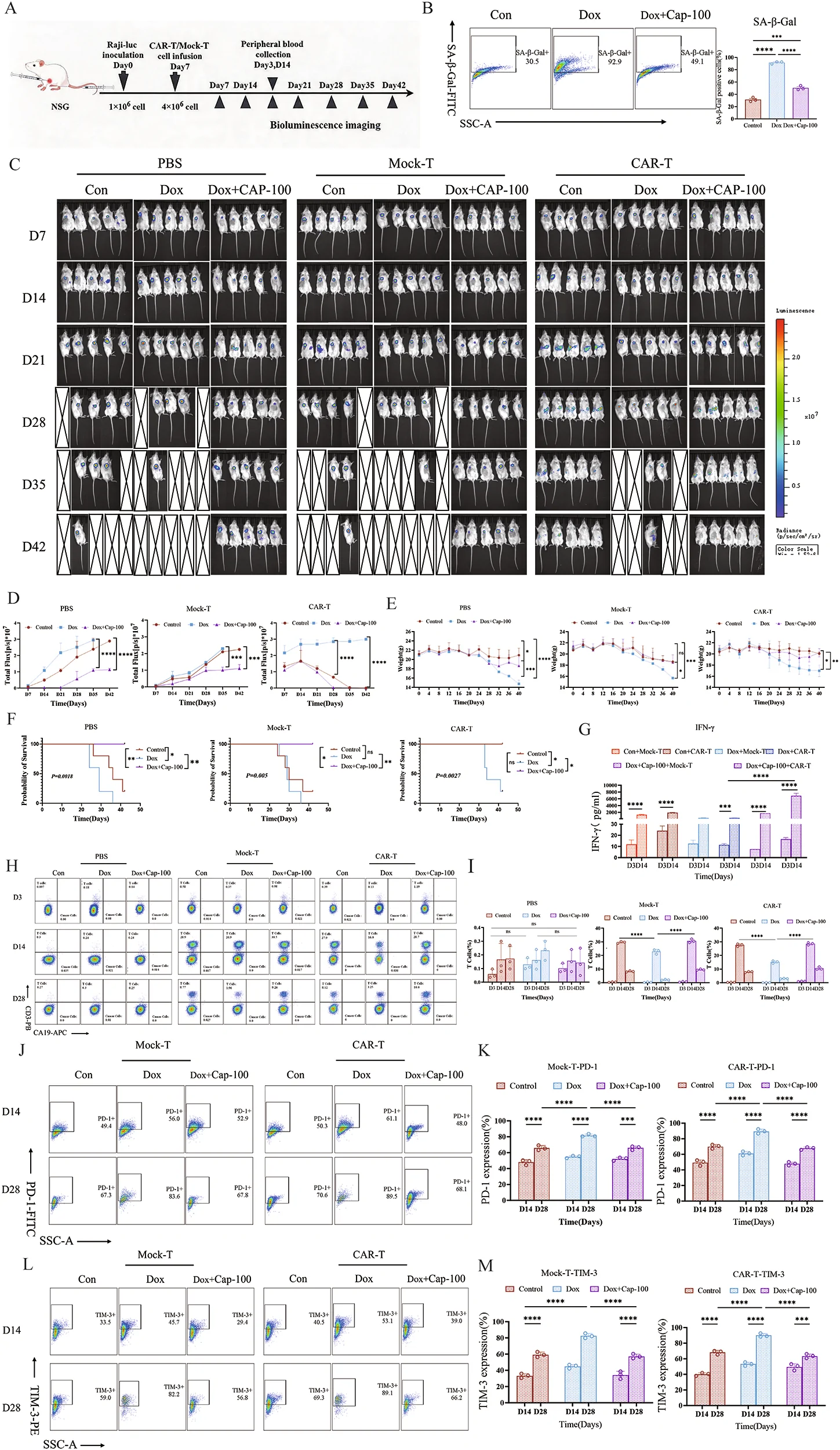
In vivo CCR7 blockade enhances CAR-T anti-tumor efficacy and reverses therapy-induced senescence. **A** Schematic diagram of the in vivo experimental protocol. **B** Flow cytometric analysis of SA-β-Gal expression in single-cell suspensions of tumor tissues from different groups. **C** Schematic diagram of in vivo imaging results. **D** Statistical Analysis of Fluorescence Intensity in In Vivo Mouse Imaging. **E** Changes in body weight in mice across groups. **F** Survival curves for mice across groups. **G** IFN-γ release in mouse serum. **H** Flow cytometric analysis of residual T cells in mouse peripheral blood at different time points following T-cell infusion. **I** Statistical graph of residual T cells in mouse peripheral blood at different time points following T-cell infusion. **J** Flow cytometric analysis of PD-1 expression, a marker of T-cell exhaustion, in residual T cells in mouse peripheral blood at different time points following T-cell infusion. **K** Statistical graph of flow cytometric analysis of PD-1 ,a marker of T-cell exhaustion on residual T cells in the peripheral blood of mice at different time points following T-cell infusion. **L** Flow cytometric analysis of TIM-3 expression, a marker of T-cell exhaustion, in residual T cells in mouse peripheral blood at different time points following T-cell infusion. **M **Statistical graph of flow cytometric analysis of TIM-3,a marker of T-cell exhaustion on residual T cells in the peripheral blood of mice at different time points following T-cell infusion

To validate the therapeutic potential of CCR7 blockade in combination with CAR-T cells, we established a subcutaneous xenograft model in NSG mice using Raji cells treated in vitro with Dox and Cap-100. The mice were randomly divided into three main groups: the PBS group, the Mock-T group, and the CAR-T group, and each of these was further subdivided into three subgroups: the Control group, the Dox group, and the Dox + Cap-100 group (Fig. 6A). Dox alone can induce significant tumor senescence, as evidenced by the increased levels of SA-β-Gal (Fig. 6B). The combination with Cap-100 significantly reduced SA-β-Gal expression, suggesting that CCR7 blockade can alleviate TIS (Fig. 6B). Compared with all other groups, the Doxorubicin + Cap-100 + CAR-T group exhibited the most significant tumor growth inhibition and the longest survival time (Fig. 6C–F). To address the long-term fate of senescent cells, we monitored tumor regrowth after treatment cessation. Mice treated with Dox alone showed rapid tumor relapse following an initial growth delay, whereas the Dox + Cap-100 + CAR-T group maintained durable tumor suppression without evidence of relapse during the observation period (Fig. 6C–F). To evaluate the role of CCR7-regulated TIS in CAR-T cell therapy for lymphoma, we analyzed the expansion, survival, and depletion of CAR-T cells in various models using flow cytometry. The Dox + Cap-100 + CAR-T group showed a significantly higher frequency of CD3 + CAR-T cells and a lower proportion of PD-1^+^TIM-3^+^ exhausted CAR-T cells compared to the CAR-T alone group (Fig. 6G, H, J–M). Moreover, cytokine analysis revealed increased IFN-γ production in CAR-T cells from the combination group (Fig. 6I). Collectively, these data confirm that CCR7 blockade synergizes with CAR-T therapy to overcome therapy-induced senescence, improve CAR-T functionality, and prevent tumor relapse.

### Cap-100 induces senescence-associated molecular changes via the CCR7-IKBα-ARHGAP18 axis, which can be reversed by GS143 and Y-27,632

This study aimed to determine whether blocking CCR7 could reverse chemotherapy-induced cellular senescence. To this end, the study was designed with four treatment groups: a control group (Con), a Dox-treated group (Dox), a Cap-100-treated group (Cap-100), and a group treated with a combination of Dox and Cap-100 (Dox + Cap-100). The expression levels of CCR7 and other typical senescence-related signalling molecules were examined using Western blot analysis in each group.

**Fig. 7:**
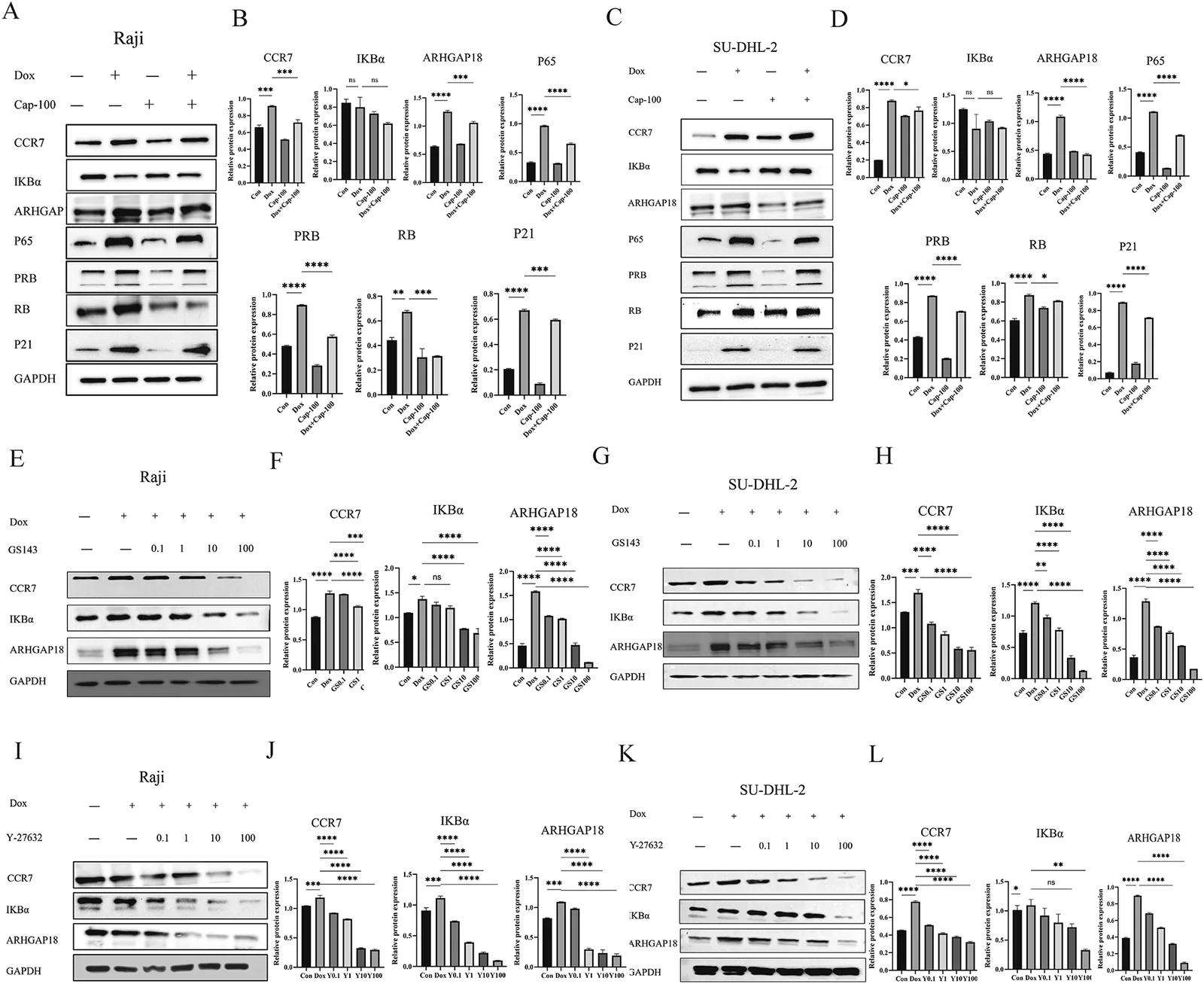
Western Blot validates that blocking CCR7 reverses Dox-induced senescence. **A** After adding Dox and Cap-100 to Raji cells for 72 h, the total amount of cell lysed proteins was collected, and then changes in senescence-associated proteins such as P21, PRB, and CCR7 were detected using Western blotting; **B** Western blotting was used to detect the changes in the senescence-associated proteins such as P21, PRB, and CCR7 after treatment of Raji cells. ImageJ for gray value reading and statistical analysis. **C** After adding Dox and Cap-100 to SU-DHL-2 cells for 72 hours, the total amount of cell lysate proteins was collected, and then changes in senescence-related proteins, P21, PRB, and CCR7, etc., were detected using Western blotting. **D** Western blotting was used to detect changes in senescence-related proteins P21, PRB, and CCR7, etc., after treatment of SU-DHL-2 cells, followed by grayscale readings using ImageJ and statistical analysis. Three replicates were used for each group. **E** Raji cells were treated with Dox for 72 hours, followed by the addition of different concentration gradients of GS143, and the changes in the expression of CCR7,IKBα,ARHGAP18 were analyzed. **F** The results of the (**E**) plot were read in grayscale values using ImageJ and analyzed statistically. **G** SU-DHL-2 cells were treated with Dox for 72 hours, followed by the addition of different GS143 with different concentration gradients, and the expression changes of CCR7,IKBα,ARHGAP18 were detected by Western Blot. **H** The results of the (**G**) plot were read in gray values using ImageJ and statistically analyzed. **I** Raji cells were treated with Dox for 72 hours, followed by the addition of Y-27632 with different concentration gradients, and the expression changes of CCR7, IKBα,ARHGAP18 were detected by Western Blot. Changes in the expression of CCR7.KMT2D.H3K9me3. **J** The results of the (**I**) graphs were read in gray values using ImageJ and statistically analyzed. **K** SU-DHL-2 cells were treated with Dox for 72 hours, followed by the addition of different concentration gradients of Y-27632, and a Western Blot was used to detect the expression of CCR7,IKBα,ARHGAP18 Changes. **L** The results of the K-plot were read in grayscale using ImageJ and statistically analyzed. *p < 0.05, **p < 0.01, ***p < 0.001, ****p < 0.0001

The results indicated that Dox treatment significantly induced cells (Raji and SU-DHL-2) to enter a senescent state. This was evidenced by the significantly increased expression of p21 and p65 along with increased levels of CCR7. Adding Cap-100 treatment on top of Dox resulted in decreased p65 expression and significantly decreased p21 expression (Fig. 7A–D). This suggests that CCR7 blockade could reverse the Dox-induced senescence phenotype and inhibit activation of the NF-κB signalling pathway. These results hint at the possibility that CCR7 plays a role in maintaining chemotherapeutic stress-induced senescence. Further verification of the key regulatory sites of the NF-κB pathway revealed that IKBα, CCR7, and ARHGAP18 expression decreased sequentially when GS143 (an IKBα inhibitor) or Y-27,632 (a ROCK pathway inhibitor) was added to Dox treatment at a concentration gradient (Fig. 7E–L). This suggests that Dox’s modulation of senescence-related signaling could be partially blocked by these two types of inhibitors. This indicates that Dox’s mechanism of action depends on CCR7-IKBα-ARHGAP18.

### CCR7–KMT2D-AFDN axis links membrane signaling to Rap1 pathway activation during senescence reversal

**Fig. 8:**
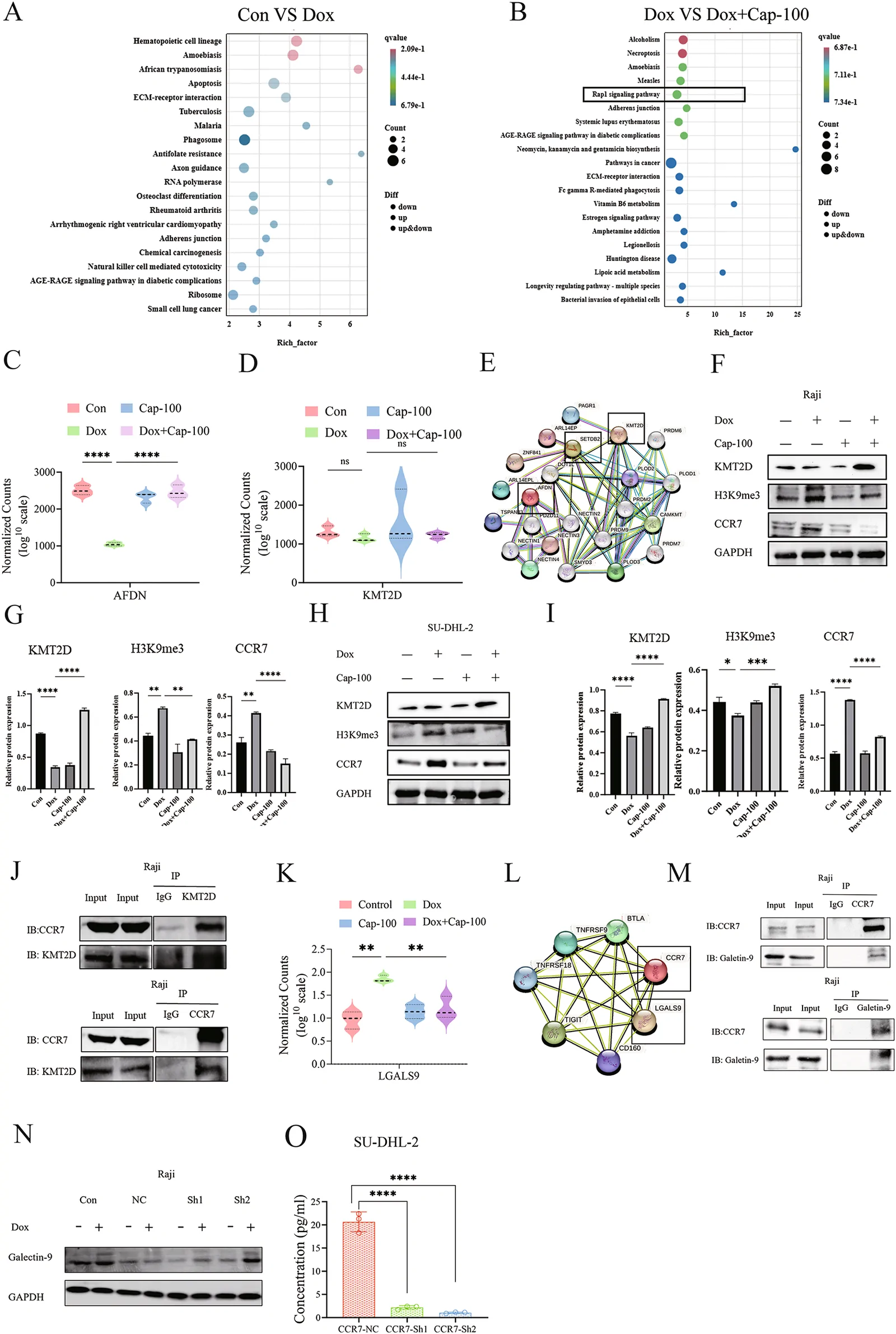
Mechanistic validation of KMT2D/H3K9me3/CCR7 in aging. **A** KEGG pathway enrichment bubble plot for differentially expressed genes (DEGs) in Raji cells (Con vs Dox). **B** KEGG pathway enrichment bubble plot for differentially expressed genes (DEGs) in Raji cells (Dox vs Dox+Cap-100). **C** Violin plot of AFDN expression across four experimental groups. **D** Violin plot of KMT2D expression across four experimental groups. **E** Protein–protein interaction (PPI) network between KMT2D, SETDB2, and AFDN visualized using STRING. **F** The control (Con), Dox, Cap-100 and Dox+Cap-100 co-treated groups of Raji cells were used to detect the expression changes of H3K9me3, KMT2D and CCR7 by Western blot. **G** The results of the (**A)**-plot were read in grayscale using ImageJ and statistically analyzed. **H** The control (Con), Dox, Cap-100 and Dox+Cap-100 co-treated groups of SU-DHL-2 cells were used to detect the expression changes of H3K9me3, KMT2D and CCR7 by Western blot. **I** The results of the (**C**) graph were read in grayscale values using ImageJ and statistically analyzed. **J** Co-IP assay and immunoblotting with CCR7/KMT2D antibody (or IgG) in Raji cells. **K** Protein–protein interaction (PPI) network between CCR7 and LGALS9 visualized using STRING. **L** Violin plot of LGALS9 expression across four experimental groups. **M** Co-IP assay and immunoblotting with CCR7/LGALS9 antibody (or IgG) in Raji cells. **N** Following knockdown of CCR7 overexpression, Dox was administered for 72 hours. Western blot analysis was performed to assess changes in Galectin-9 protein levels. **O** Following knockdown of CCR7 overexpression, ELISA detection of Galectin-9 release. *p < 0.05, **p < 0.01, ***p < 0.001, ****p < 0.0001

To further elucidate the regulatory mechanism by which the C-C chemokine receptor 7 (CCR7) antagonist Cap-100 modulates Dox-induced cellular senescence and reduces exhaustion, we performed transcriptome sequencing analysis on the control group and the following groups: Dox group, Cap-100 group, and Dox + Cap-100 group. Regarding induced cellular senescence, results demonstrated that Dox treatment significantly inhibited the Rap1 signalling pathway, whereas co-administration of Cap-100 reactivated this pathway (Fig. 8A–B). The key Rap1 pathway factor AFDN exhibited downregulated expression in the Dox group; this gene expression was regulated, and following Cap-100 treatment, its expression levels recovered to near-control group levels (Fig. 8C), although the difference was not statistically significant due to the presence of individual outliers. The KMT2D gene exhibited a similar trend, albeit with a smaller magnitude of change (Fig. 8D). The STRING database [13] predicted potential interactions between AFDN, SETDB2, and KMT2D (Fig. 8E), suggesting CCR7 may mediate cellular senescence by regulating the Rap1 signalling pathway and the AFDN-KMT2D axis. Western blot analysis revealed that Dox treatment significantly upregulated CCR7 and H3K9me3 expression while downregulating KMT2D expression, an effect effectively reversed by Cap-100 (Fig. 8F–I). Co-immunoprecipitation confirmed direct protein interaction between CCR7 and KMT2D (Fig. 8J). This study indicates that CCR7 may regulate cellular senescence by modulating the AFDN-KMT2D and Rap1 signalling pathways, forming a signalling axis linking membrane receptor signalling, epigenetic regulation, and Rap1 pathway activation to jointly govern senescent processes. Regarding the alleviation of T-cell exhaustion, the violin plot from transcriptome sequencing revealed markedly elevated expression of LGALS9 (Galectin-9) in the Dox group, whereas this expression returned to control levels in the Dox + Cap-100 group (Fig. 8K). Online STRING analysis (Fig. 8L) and co-immunoprecipitation (Fig. 7M) results confirmed the interaction between CCR7 and LGALS9 (galectin-9). Following lentiviral transduction to knock down or overexpress CCR7 in Raji and SU-DHL-2 cells (Additional file 2:S4), Galectin-9 expression was assessed (Fig. 8N and O). Results demonstrated that Galectin-9 levels fluctuated in tandem with CCR7 alterations, indicating CCR7 functions as an upstream regulator of Galectin-9 in this process. Dox-induced cellular stress ageing elevates CCR7 expression, subsequently increasing downstream Galectin-9 expression to form an immunosuppressive tumour microenvironment. Its binding to TIM-3 on T cells causes CAR-T cell exhaustion and reduces their cytotoxic efficacy. Co-administration of Cap-100 blocks Galectin-9 release, preventing immunosuppressive tumour microenvironment formation. This alleviates T cell exhaustion, enabling them to function more effectively.

## Discussion

Relapse and therapeutic resistance remain the most formidable challenges in the management of relapsed/refractory B-NHL [14, 15]. TIS, a state of persistent cell cycle arrest triggered by chemotherapy, has long been recognized as a double-edged sword in cancer treatment [16, 17, 18]. While it acutely restrains tumor expansion, senescent cells can acquire stem-like properties, secrete a pro-inflammatory and immunosuppressive SASP, and foster a microenvironment that drives long-term relapse [19,20,21]. Therefore, identifying key regulators of TIS is crucial for developing novel strategies to overcome therapeutic failure. Our study identifies the chemokine receptor CCR7, traditionally associated with immune cell homing and metastasis, as a key regulator of TIS in B-NHL with significant impact on both chemotherapy and immunotherapy efficacy.

TIS is characterized by markers including SA-β-Gal activity, elevated H3K9me3, and induction of p21 [22,23,24]. We show that chemotherapy induces senescence in B-NHL cells, accompanied by a marked upregulation of CCR7, which is critical for maintaining the senescent state. Mechanistically, CCR7 modulates TIS through the IκBα–NF-κB signaling axis. Specifically, CCR7 upregulation suppresses IκBα, resulting in sustained NF-κB activation, a key feature of TIS in hematologic malignancies, which in turn drives the expression of the cell cycle inhibitor p21 and SASP factors. Importantly, blockade of CCR7 with Cap-100 restores IκBα levels, attenuates NF-κB activation, and reverses the senescent phenotype, highlighting CCR7 inhibition as a potential therapeutic approach to counteract chemotherapy-induced senescence in B-NHL.

Lymphoma cells survive therapeutic stress by exploiting plasticity between states like senescence and stemness. Our results establish CCR7 as a central regulator of this adaptability. Extending prior associations of high tumor CCR7 with poor prognosis [11], we show it sustains TIS to evade apoptosis, upregulates resistance-conferring stemness markers, and promotes a tumorigenic SASP that remodels the microenvironment. Conversely, CCR7 blockade reverses the TIS and stem-like phenotype in lymphoma cells, consequently enhancing their chemosensitivity. Stem-like cells are a well-known reservoir of relapse in B-NHL, as they resist conventional therapies and repopulate the tumor bulk [8]. Additionally, our results revealed that Cap-100 enhances Dox-induced apoptosis and suppresses proliferation, further supporting CCR7 as a target to eliminate therapy-resistant subpopulations. Notably, the effect we observed is specific to B‑NHL cells. In contrast to broad CCR7-targeting strategies, which may impair immune cell trafficking, our approach focuses on targeting CCR7 on tumor cells. CCR7 expression on T cells from DLBCL patients was significantly lower than that of healthy controls, and those with poor response to CAR-T therapy also have lower CCR7 expression, underscoring the importance of sparing T cell CCR7. Our findings are consistent with reports that CCR7 + T cells retain stem cell properties and effector functions, marking high-quality anti-tumor immunity [25], but high CCR7 expression on lymphoma cells is significantly associated with poor patient prognosis [11]. We believe that CCR7 is a functional marker of healthy T cells, but during repeated chemotherapy, it may be exploited by B-NHL cells to enhance their survival and induce immunosuppressive senescence programs. Therefore, selectively inhibiting CCR7 signaling in tumor cells would not impair the function of CCR7 + T cells. This contrasts with pan-CCR7 inhibition, which might impair T cell homing to tumors, highlighting the precision of our strategy. Our longitudinal study further supports clinical relevance, as CCR7 pathway factors (IκBα, CCL21) correlate with CAR-T efficacy, with low IκBα and high CCL21 in PD/r/r patients indicating a more immunosuppressive TME. Cap-100, by normalizing these factors, could tip the balance toward effective immune clearance. Therefore, tumor cell-specific CCR7 blockade avoids compromising T cell fitness, a key advantage for combination therapies.

CAR-T cell therapy has revolutionized relapsed/refractory B-NHL treatment, but its efficacy is limited by immune evasion, driven by both tumor cell intrinsic resistance and an immunosuppressive TME [15, 26]. Our study uncovers that reversal of CCR7-driven B-NHL-TIS can synergistically enhance the cytotoxicity of CAR-T cells against lymphoma cells and reduce CAR-T cell exhaustion. First, TIS cells secrete SASP factors, which recruit immunosuppressive MDSCs and Treg cells and dampen CAR-T activity [27]. Second, during TIS conditions, cells release LGALS9 (Galectin-9), which binds to TIM-3 on the surface of T cells, guiding them toward exhaustion. This ultimately leads to disease recurrence and diminished CAR-T efficacy. By reversing TIS, Cap-100 reduces SASP, alleviating TME suppression. Third, CCR7 maintains stem-like markers on lymphoma cells, which are associated with reduced CAR-T recognition. shCCR7 downregulates these markers, enhancing tumor cell susceptibility to CAR-T-mediated killing. Here we have shown in a subcutaneous B-NHL xenograft model that targeting CCR7-driven TIS synergizes with CAR-T therapy to achieve long-term tumor control. CCR7 blockade alone reduced in vivo senescence markers and SASP factors, while the addition of CAR-T cells further reversed the senescent phenotype and promoted durable suppression. Moreover, CCR7 blockade enhanced CD3^+^T cell infiltration, reduced PD-1^+^TIM-3^+^ exhausted CAR-T cells, and preserved IFN-γ production, consistent with our in vitro observations. CCR7-driven TIS creates an immunosuppressive niche that blunts CAR-T function, and reversing TIS restores anti-tumor immunity. Combining chemotherapy, CCR7 blockade, and CAR-T cell therapy achieved durable tumor suppression without relapse. Our data provide the first evidence that targeting therapy-induced senescence in B-NHL can overcome CAR-T resistance. This strategy may help improve outcomes in relapsed/refractory B-NHL patients where CAR-T therapy currently fails.

To further explore the potential mechanisms underlying CCR7-driven B-NHL-TIS, a major advance of our study is the identification of a direct interaction between CCR7 and KMT2D, and its link to Rap1 signaling. KMT2D is a histone H3K4 methyltransferase that is known to maintain chromatin accessibility and transcriptional activity, and its loss in cancer leads to epigenetic silencing of tumor suppressors[28]. During chemotherapy-induced TIS, CCR7 is activated and participates in the maintenance of senescence. During this process, KMT2D expression is inhibited, leading to the accumulation of H3K9me3 and ultimately maintaining the TIS phenotype. KMT2D is a key enzyme that regulates chromatin openness, and its downregulation may lead to aging-related epigenetic reprogramming [29]. At the same time, H3K9me3 is a marker of heterochromatin formation [30], and its high expression is consistent with the core characteristics of TIS. After Cap-100 blocks CCR7, the above process can be reversed; KMT2D is restored, and H3K9me3 is reduced, leading to apoptosis of lymphoma cells. This provides the most direct molecular evidence for targeting CCR7 to reverse lymphoma TIS and enhance therapeutic sensitivity. Co-IP confirmed a direct interaction between CCR7 and KMT2D, suggesting that CCR7 may sequester or degrade KMT2D to enforce epigenetic silencing. Furthermore, transcriptome sequencing revealed that the Rap1 signaling pathway, which is critical for cell adhesion, survival, and T cell activation, is significantly activated in the Dox + Cap-100 group. AFDN, a Rap1 effector, is downregulated in B-NHL-TIS but restored by Cap-100, likely via KMT2D-mediated transcription. This suggests that the CCR7-KMT2D-AFDN axis connects epigenetic regulation to Rap1-dependent survival. In senescent cells, CCR7 suppresses KMT2D, reducing AFDN and Rap1 activity. Cap-100 reverses this, reactivating Rap1 to promote apoptosis. This axis is novel in the biology of B-NHL-TIS. While Rap1 has been linked to cancer cell migration, its role in senescence reversal is unreported. Our findings position KMT2D as a critical epigenetic mediator of CCR7 signaling, opening new avenues to target senescence via epigenetic modulators in combination with CCR7 blockade, not previously described in B-NHL. Additionally, this study innovatively demonstrates that blocking CCR7 enhances CAR-T therapy efficacy by reducing LGALS9, providing meaningful guidance for subsequent clinical translation.

The biological significance of CCR7-dependent TIS regulation is multifaceted. Senescent cells resist apoptosis while promoting malignancy through SASP [18]. By sustaining TIS, CCR7 enables B-NHL cells to survive chemotherapy, retain stem-like properties, and seed relapse. This explains why CCR7 knockdown in Raji cells reduced stemness markers and why Cap-100 enhanced apoptosis, as CCR7 blockade disrupts the senescent “survival program,” forcing cells to exit TIS and undergo cell death.

Our work redefines CCR7 as a key regulatory target of B-NHL-TIS, not just a leukocyte migration marker, which has important clinical significance. In patients with relapsed or refractory B-NHL, Cap-100 can reverse TIS and convert stem-like lymphoma cells that are resistant to chemotherapy into sensitive cells susceptible to apoptosis. This reduces the risk of refractoriness and relapse. Treating B-NHL-TIS lymphoma cells with Cap-100 can improve the efficacy of CAR-T therapy by reducing TME inhibition and tumour cell stemness. This is particularly valuable in cases of relapsed or refractory B-NHL, where CAR-T therapy failure is common. T-cell CCR7 expression can be used as a predictive marker for CAR-T therapy response, while tumour CCR7 and KMT2D levels may help to identify patients who could benefit from CCR7-targeted therapy. However, this study’s main limitation is the need for further in vivo verification of the synergistic effect of Cap-100 and CAR-T, and exploration of whether CCR7 blockade affects other immune cells.

In conclusion, we revealed that CCR7 regulates B-NHL-TIS via KMT2D-Rap1 for the first time and clarified the molecular mechanism by which lymphoma cells evade chemotherapy. Blocking the CCR7 receptor on tumour cells can reverse TIS and eliminate tumour stem cell-like characteristics. It can also enhance the killing activity of CAR-T cells while preserving the anti-tumour function of T cells. Using CCR7/TIS as a new intervention target offers a breakthrough approach to the chemotherapy-immunotherapy of relapsed and refractory B-NHL. This is expected to overcome treatment resistance by reshaping tumour phenotype and the immune microenvironment.

## Conclusions

Dox‑induced TIS in B‑NHL is driven by CCR7 upregulation, which activates NF‑κB, suppresses Rap1/AFDN‑KMT2D, and increases Galectin‑9 secretion, promoting SASP and CAR‑T exhaustion via TIM‑3/PD‑1 pathways. CCR7 blockade with Cap‑100 reverses senescence, enhances apoptosis, restores Rap1 signaling, reduces Galectin‑9 release, and alleviates T‑cell exhaustion, thereby improving CAR‑T cytotoxicity in vitro and in vivo, with sustained tumor control and prolonged survival. Clinically, low CCR7 on patient T cells correlates with poorer survival and treatment failure. Thus, CCR7 is a central mediator of TIS‑related immune evasion, a promising therapeutic target to sensitize tumors to CAR‑T therapy, and a potential biomarker for clinical outcome.

## Supplementary Information

Below is the link to the electronic supplementary material.

Supplementary Material 1: Tables S1-S2

Supplementary Material 2: Figures S1-S4

Supplementary Material 3: Original uncropped blots/gels. Original, uncropped images of all Western blot and Co-immunoprecipitation (Co-IP) membranes presented in the study

## Data Availability

The sequencing data generated in this study have been deposited in the National Center for Biotechnology Information (NCBI) database (DataView: https://dataview.ncbi.nlm.nih.gov/object) under the BioProject number PRJNA1375446. The corresponding BioSample accession identifiers are from SAMN53685632 to SAMN53685642. The STRING database: Szklarczyk D, Kirsch R, Koutrouli M, Nastou K, Mehryary F, Hachilif R, Gable AL, Fang T, Doncheva NT, Pyysalo S, Bork P, Jensen LJ, von Mering C. The STRING database in 2023: protein-protein association networks and functional enrichment analyses for any sequenced genome of interest. Nucleic Acids Res. 2023 Jan 6;51(D1): D638-D646. doi: 10.1093/nar/gkac1000. PMID: 36370105; PMCID: PMC9825434.Additional data are provided in the supplementary materials or are available from the corresponding author upon reasonable request.

## Abbreviations

CAR-T: Chimeric Antigen Receptor T-cell
B-NHL: B-cell non-Hodgkin lymphoma
TIS: Therapy-Induced Senescence
Dox: doxorubicin
SASP: senescence-associated secretory phenotype
CCR7: C-C Chemokine Receptor 7
SA-β-Gal: Senescence-Associated β-Galactosidase
H3K9me3: H3 lysine 9 trimethylation
TMEs: tumor microenvironments
PD: progressive disease
ND: new diagnosis
r/r: relapse/refractory
PFS: progression-free survival
OS: overall survival
DLBC: diffuse large B-cell lymphoma
CLL: chronic lymphocytic leukemia
MCL: mantle cell lymphoma
LPL: lymphoplasmacytic lymphoma
MALT: mucosa-associated lymphoid tissue lymphoma
