# Supplementary material for: Targeting CCR7-KMT2D enhances CAR-T cell efficacy by suppressing therapy-induced senescence in B-cell non-Hodgkin lymphoma
Source: BMC Med. 2026 Jun 26;24:492. doi: 10.1186/s12916-026-05030-4 (PMC13579993; doi:10.1186/s12916-026-05030-4)
Supplement: Supplementary file 1 [file 12916_2026_5030_MOESM1_ESM.docx]

**Table2.** ELISA kits used in this study

| Target | Company | Catalog Number | Country |
| --- | --- | --- | --- |
| CCL19 | RUIXINBIO | RX102250H | China |
| SLAMF1 | RUIXINBIO | RX107094H | China |
| CD34 | RUIXINBIO | RX104490H | China |
| TGF-β1 | RUIXINBIO | RX104788H | China |
| IκBα | RUIXINBIO | RX105828H | China |
| CCR7 | RUIXINBIO | RX100007H | China |
| LGR5 | RUIXINBIO | RX100977H | China |
| IL-35 | RUIXINBIO | RX106132H | China |
| CCL21 | RUIXINBIO | RX105345H | China |
| CD44 | RUIXINBIO | RX106389H | China |

**Table 3:** List of primary antibodies used in this study

| **Antibody** | **Dilution Ratio** | **CatalogNumber** | **Company** |
| --- | --- | --- | --- |
| CCR7 | 1:1,000 | A0121 | ABclonal |
| P21 | 1:2,000 | #2947 | CellSignalingTechnology(CST) |
| PRB | 1:2,000 | #8516 | CellSignalingTechnology(CST) |
| RB | 1:2,000 | #9309 | CellSignalingTechnology(CST) |
| IκBα | 1:5,000 | ab32518 | Abcam |
| H3K9me3 | 1:5,000 | ab8898 | Abcam |
| ARHGAP18 | 1:2,000 | ab262926 | Abcam |
| KMT2D | 1:2,000 | ab126951 | Abcam |
| LGALS9 | 1:2000 | 17938-1-AP | proteintech |

The primary antibodies utilized for Western blotting and immunodetection are enumerated in Table 3. Antibodies were diluted in 5% skim milk or BSA according to the manufacturer's instructions. All antibodies were validated and widely used in previous studies.
