# Supplementary material for: Targeting CCR7-KMT2D enhances CAR-T cell efficacy by suppressing therapy-induced senescence in B-cell non-Hodgkin lymphoma
Source: BMC Med. 2026 Jun 26;24:492. doi: 10.1186/s12916-026-05030-4 (PMC13579993; doi:10.1186/s12916-026-05030-4)
Supplement: Supplementary file 2 [file 12916_2026_5030_MOESM2_ESM.docx]

Fig S1:Cytokine Expression Following CCR7 Intervention in Raji Cells

Fig. S2: CCR7 expression in Raji and SU-DHL-2 cells at different time points under varying treatment conditions, alongside changes in cell density and diameter during culture

(A) Flow cytometry to detect the proportion of CCR7 in Raji cells at different time points under different treatment conditions. (B) Statistical graph of the proportion of CCR7 in Raji cells detected by flow cytometry. (C) Cell density of Raji cells at different time points under varying treatment conditions. (D) Diameter of Raji cells at different time points under varying treatment conditions. (E)Flow cytometry to detect the proportion of CCR7 in SU-DHL-2 cells at different time points under different treatment conditions. (F)Statistical graph of the proportion of CCR7 in SU-DHL-2 cells detected by flow cytometry. (G) Cell density of Raji cells at different time points under varying treatment conditions. (H) Diameter of Raji cells at different time points under varying treatment conditions.

FigS3: Expression of serum cytokines in patients receiving CD19 CAR-T

FigS4: Raji/SU-DHL-2 lentiviral transfection for knockdown and overexpression of CCR7, validated by qPCR

(A) Observation of CCR7 lentiviral transduction in Raji cells under fluorescence microscopy. (B) qPCR validation of CCR7 knockdown and overexpression in Raji cells via lentiviral infection. (C) Observation of CCR7 lentiviral transduction in SU-DHL-2 cells under fluorescence microscopy. (D) qPCR validation of CCR7 knockdown and overexpression in SU-DHL-2 cells via lentiviral infection.
